# Assessment of glucose regulation in pregnancy after gastric bypass surgery

**DOI:** 10.1007/s00125-017-4437-6

**Published:** 2017-09-16

**Authors:** Christian S. Göbl, Latife Bozkurt, Andrea Tura, Michael Leutner, Laura Andrei, Lukas Fahr, Peter Husslein, Wolfgang Eppel, Alexandra Kautzky-Willer

**Affiliations:** 1 Department of Obstetrics and Gynecology, Division of Obstetrics and Feto-maternal Medicine, Medical University of Vienna, Vienna, Austria; 2 Department of Internal Medicine III, Division of Endocrinology and Metabolism, Gender Medicine Unit, Medical University of Vienna, Waehringer Guertel 18–20, A-1090 Vienna, Austria; 3 Metabolic Unit, Institute of Neuroscience, National Research Council, Padova, Italy

**Keywords:** Fetal development, Gastric bypass, Insulin secretion, Insulin sensitivity, Pregnancy

## Abstract

**Aims/hypothesis:**

Roux-en-Y gastric bypass (RYGB) surgery is characterised by glycaemic variability. Prospective studies of glucose metabolism in pregnancy after RYGB are not available, therefore this study aimed to evaluate physiological alterations in glucose metabolism in pregnancy following RYGB.

**Methods:**

Sixty-three pregnant women (25 who underwent RYGB, 19 non-operated obese control women and 19 normal weight control women) were included. Frequently sampled 3 h OGTTs and 1 h IVGTTs were performed between 24 and 28 weeks of gestation and, in a subgroup, were repeated at 3–6 months after delivery.

**Results:**

We observed major alterations in glucose kinetics during the OGTT, including an early increase in plasma glucose followed by hypoglycaemia in 90% of women who had previously undergone RYGB. The higher degree of glycaemic variability in this group was accompanied by increased insulin, C-peptide and glucagon concentrations after oral glucose load, whereas no differences in insulin response were observed after parenteral glucose administration (RYGB vs normal weight). IVGTT data suggested improved insulin sensitivity (mean difference 0.226 × 10^−4^ min^−1^ [pmol/l]^−1^ [95% CI 0.104, 0.348]; *p* < 0.001) and disposition index in pregnancies after RYGB when compared with obese control women. However, subtle alterations in insulin action and beta cell function were still observed when comparing women who had undergone RYGB with the normal-weight control group. Moreover, we observed that fetal growth was associated with maternal glucose nadir levels and insulin secretion in offspring of those who had previously undergone RYGB.

**Conclusions/interpretation:**

Pregnancies after RYGB are affected by altered postprandial glucose, insulin and C-peptide dynamics. Insulin sensitivity is improved by RYGB, although subtle alterations in beta cell function are observed. Longitudinal studies are needed to assess potential consequences for fetal development and pregnancy outcomes.

**Electronic supplementary material:**

The online version of this article (10.1007/s00125-017-4437-6) contains peer-reviewed but unedited supplementary material, which is available to authorised users.

## Introduction

The obesity epidemic has become a major healthcare problem in industrialised and developing countries and is strongly associated with reduced health-related quality of life [1] and an increased risk for metabolic and cardiovascular disorders [2]. Bariatric surgery is recommended as a possible treatment for morbid obesity if conservative interventions fail to achieve satisfactory weight reduction [3, 4]. Hereby, Roux-en-Y gastric bypass (RYGB) is the most commonly performed technique and has long-lasting effects regarding weight loss (up to 30%) and prevention or improvement of type 2 diabetes [5, 6].

An increase in the prevalence of obesity is already being seen in younger populations [7], meaning that weight-loss surgery is emerging as a treatment option even in women of reproductive age. Previous retrospective reports and register studies have addressed pregnancy complications and pregnancy outcomes following gastric bypass and have demonstrated beneficial effects such as reduced incidence of pre-existing and gestational diabetes mellitus (GDM) [8, 9], possibly contributing to a lower prevalence of large for gestational age (LGA) offspring [10–12]. However, a marked increase in the prevalence of small for gestational age (SGA) neonates, along with a small increase in the rates of stillbirths or neonatal deaths, indicates a higher risk for perinatal morbidity and mortality [13, 14]. In addition, studies in non-pregnant women suggest that gastric bypass surgery is also characterised by an exaggerated postprandial rise in blood glucose followed by hyperinsulinaemic hypoglycaemia, despite long-term improvements in type 2 diabetes onset or management [15]. These effects might also occur during gestation, with possible implications for pregnancy and GDM screening. However, data from detailed examination of glucose metabolism in pregnancies following RYGB surgery are currently not available.

This study aims to evaluate physiological changes in glucose regulation in pregnant women with a history of gastric bypass surgery, including dynamic measurements of glucose, insulin, C-peptide and glucagon after oral glucose stimulation as a primary objective. Therefore, variables of glucose disposal (insulin sensitivity, insulin secretion and beta cell function representing the major components of altered glucose metabolism) were examined during pregnancy and after delivery. Frequently sampled OGTT and IVGTT were used to quantify gut-dependent and gut-independent effects. These variables were compared between mothers who had undergone RYGB surgery and two control groups (a normal weight and an obese group). Changes in glucose regulation after pregnancy and associations between maternal glucose metabolism and neonatal weight were assessed as secondary objectives.

## Methods

### Study participants and experimental methods

Participants were consecutively recruited among visitors to our pregnancy outpatient department (Department of Obstetrics and Gynecology, Division of Obstetrics and Feto-maternal Medicine, Medical University of Vienna) between April 2014 and February 2016. A total of 63 women participated in the study. Participants included 25 women with a history of RYGB, 19 non-operated obese women (pre-conceptional BMI ≥ 35 kg/m^2^) and 19 normal weight control women (normal glucose tolerant and pre-conceptional BMI < 25 kg/m^2^). The calculation of pre-conceptional BMI was based on self-reported pre-gestational weight. The median time (IQR) after RYGB surgery was 3.3 years (2.3–5.7). Women who received insulin or glucose-lowering drugs at time of recruitment were excluded.

To further control for possible bias owing to differences in status of overweight or obesity, some comparisons were repeated after matching the RYGB and control groups; we used 1:1 nearest-neighbour matching (with a caliper of 0.25 SD of the propensity score) to create two groups (14 matched pairs) that were optimally balanced for BMI at the first visit [16]. The BMI-matched group contained *n* = 6 non-operated obese and *n* = 8 normal weight control women. Since there were fewer study participants in both control groups, matching observations were not found for the remaining *n* = 11 women in the RYGB group. Additional inclusion of the participant’s age into the matching algorithm did not substantially improve the balance of the covariates and was therefore omitted.

**Fig. 1:**
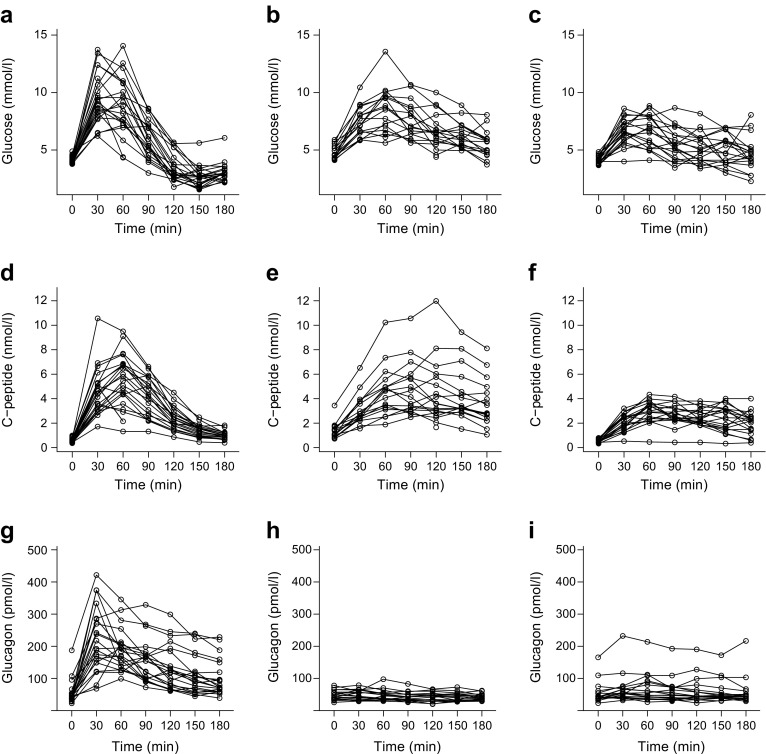
Spaghetti plots of plasma glucose (**a**–**c**), C-peptide (**d**–**f**) and glucagon kinetics (**g**–**i**) during a 3 h OGTT in pregnant women after RYGB (**a**, **d**, **g**) and in severely obese (BMI ≥ 35 mg/m^2^) (**b**, **e**, **h**) and normal weight (BMI < 25 mg/m^2^) (**c**, **f**, **i**) pregnant women

A detailed metabolic characterisation of the study population was performed between 24 and 28 weeks of gestation and included routine laboratory assessments and anthropometric measurements in addition to an extended 3 h 75 g OGTT after at least 8 h of fasting with measurements of glucose, insulin, C-peptide and glucagon (at 0, 30, 60, 90, 120, 150 and 180 min). In a short, frequently sampled IVGTT performed on a second study day, glucose (300 mg/kg body weight) was infused for 30 s, starting at time 0, and insulin (0.05 U/kg) was infused from 20 to 25 min. Plasma concentrations of glucose, insulin and C-peptide were assessed at fasting as well as at 3, 4, 5, 6, 8, 10, 15, 20, 30, 40, 50 and 60 min after glucose infusion. The experiments were repeated 3–6 months after delivery. At the postpartum examination, 15 women reported that they breastfed their infants (RYGB, *n* = 4; obese, *n* = 3; normal weight, *n* = 8) and four used hormonal contraceptives (RYGB, *n* = 0; obese, *n* = 1; normal weight, *n* = 3). A detailed overview of missing examinations (OGTT or IVGTT or both) is provided in the electronic supplementary material (ESM) Fig. 1.

Sex- and age-adjusted birthweight percentiles of the Austrian population were assessed using local growth standard curves. After excluding data from nine women with multiple pregnancies (RYGB, *n* = 5; obese, *n* = 3; normal weight, *n* = 1) and six women without this data, as they gave birth at another institution, neonatal biometry was available in a subgroup of 48 women (RYGB, *n* = 16; obese, *n* = 16; normal weight, *n* = 16).

This study was approved by the Ethics Committee of the Medical University of Vienna and was performed in accordance with the Declaration of Helsinki. All participants gave written informed consent to participate in this study.

### Calculations and laboratory methods

Dynamic measurements during OGTT and IVGTT are presented as mean values. The shape index of glucose (WHOSH-G), representing the variability of plasma glucose, was calculated as the discrete second order derivate of the 3 h OGTT glucose curve. This index was calculated for all study participants and then normalised to the average (mean value) of the index itself in the whole dataset (i.e. 2.24 × 10^−3^ mmol/l min^−2^) [17]. Insulin sensitivity in fasting conditions was described using the quantitative insulin sensitivity check index (QUICKI), representing an approximation of hepatic insulin action [18]. Whole-body insulin sensitivity from the IVGTT was examined using the calculated sensitivity index (CSI) [19], which is a reliable surrogate of clamp insulin sensitivity. Insulin secretion from the IVGTT was assessed by the mean incremental short-term insulin response 3–10 min after i.v. glucose load (acute insulin response to glucose [AIR*g*]) [20]. The disposition index (DI) was calculated as the product of CSI (× 10^−4^ min^−1^ [pmol/l]^−1^) and AIR*g* (pmol/l) to provide an estimation of beta cell function (i.e. the ability of beta cells to adapt for insulin resistance). Insulin sensitivity was additionally estimated from OGTT data by using the oral glucose insulin sensitivity index (OGIS, calculated from the first 120 min) [21] and modified insulinogenic indices were used to describe early insulin secretion (AUC_insulin_/AUC_glucose 0–60 min_), late insulin secretion (AUC_insulin_/AUC_glucose 60–180 min_) and total insulin secretion (AUC_insulin_/AUC_glucose 0–180 min_) from post-hepatic measurements [22].

All laboratory variables were measured at our certified Department of Medical and Chemical Laboratory Diagnostics (www.kimcl.at, accessed 15 August 2017) according to international standard laboratory methods. Plasma glucose concentrations were measured by the hexokinase method with a CV of 1.3%. Insulin and C-peptide were measured by chemiluminescence immune assays with CVs of 4–7% and 3–4%, respectively.

### Statistical analysis

Categorical variables were expressed as counts and percentages and compared by Fisher’s exact test. Continuous variables were expressed as mean ± SD or as median (IQR). Comparisons of continuous variables between three groups were performed by ANOVA as well as the Fisher’s protected least significant for difference tests. Rank-based inference was used in the case of skewed distributed variables and comparisons with restricted sample size were made according to Brunner and Munzel [23] and Neubert and Brunner [24]. Associations between continuous variables were examined by Spearman’s rank correlation as well as linear and nonlinear regression. Variables measured during pregnancy and after delivery in the RYGB participants were compared by a paired rank test. Analyses of change scores were additionally used to assess differences between pregnancy and postpartum. If single measurements of OGTT and IVGTT were missing (< 50% of measurements in the respective experiment), multivariate imputations by chained equations were performed to estimate the missing values by the average of m = 50 complete datasets. Details of data imputations are provided in the ESM Methods.

Statistical analysis was performed with R (V3.1.1, Vienna, Austria) and contributing packages, specifically ‘mice’ for imputations, ‘matchit’ for creating the BMI-matched control group, ‘gmodels’, ‘nparcomp’, ‘rms’ for data analyses and ‘lattice’ and ‘beeswarm’ for visualisations [25]. A two-sided *p* value ≤ 0.05 was considered statistically significant. Owing to the explorative character of this observational study, we used no further adjustment for multiple statistical testing, unless otherwise indicated.

## Results

### Baseline characteristics

Table 1 lists the participants’ characteristics and comparisons of glucometabolic variables. Despite significant weight reduction after gastric bypass (BMI changed from 46.8 kg/m^2^ to 28.6 kg/m^2^), mothers who had undergone RYGB showed a high amount of overweight (39%, BMI ≥ 25 kg/m^2^ to < 30 kg/m^2^), obesity (22%, BMI ≥ 30 kg/m^2^ to < 35 kg/m^2^) and severe obesity (13%, BMI ≥ 35 kg/m^2^). Weight gain until 24–28 weeks of gestation was comparable between the three groups (RYGB, 9.2 ± 6.1 kg vs severe obesity, 8.6 ± 7.0 kg vs normal weight, 9.4 ± 3.3 kg; *p* = 0.888), resulting in a BMI at the first study visit of 32.0 ± 5.2 kg/m^2^, 41.1 ± 4.3 kg/m^2^ and 25.1 ± 2.5 kg/m^2^ for the RYGB, severely obese and normal weight groups, respectively.

**Table 1:** Baseline characteristics and glucometabolic variables during pregnancy in women after RYGB and in control women with pre-gestational obesity (obese control group) and normal weight (normal weight control group)

Characteristic	*n*	RYGB	*n*	Obese control	*n*	Normal weight control
Age, years	25	31.9 ± 7.0	19	31.9 ± 4.8	19	30.8 ± 5.7
Parity	25	1 (0–3)	19	1 (1–2)	19	1 (0–1)
GDM history, *n* (%)	25	2 (8.0)	19	5 (26.3)	0	0 (0.0)
BMIPG, kg/m^2^	23	28.6 ± 5.0	19	38.0 ± 2.9*	19	21.7 ± 2.1*
Age at surgery, years	23	28.0 ± 6.6	–	–	–	–
BMI before surgery, kg/m^2^	18	46.8 ± 7.6	–	–	–	–
QUICKI	24	0.37 ± 0.03	19	0.31 ± 0.03*	19	0.38 ± 0.04
OGIS, ml min^−1^ m^−2^	21	517.9 ± 68.2	17	353.0 ± 86.5*	19	508.2 ± 62.3
Insulin secretion during OGTT, pmol/mmol^a^						
Early^b^	22	106 (76–135)	17	106 (74–132)	19	49 (43–81)*
Late^c^	21	57 (43–97)	17	119 (79–176)*	19	54 (40–79)
Total^d^	21	85 (57–119)	17	107 (83–152)	19	51 (42–83)*
Fasting glucose, mmol/l	24	4.15 ± 0.29	19	4.78 ± 0.56*	19	4.16 ± 0.35
Glucose during OGTT, mmol/l						
Maximum	21	9.95 ± 2.33	17	8.71 ± 1.84*	19	7.01 ± 1.24*
Mean	21	5.38 ± 0.92	17	6.73 ± 1.05*	19	5.38 ± 0.90
Fasting insulin, pmol/l^a^	24	47 (35–56)	19	132 (105–176)*	19	51 (32–69)
Insulin during OGTT, pmol/l^a^						
Maximum	21	1150 (892–1875)	17	1226 (885–2143)	19	603 (436–732)*
Mean	21	391 (299–619)	17	649 (495–1138)*	19	332 (193–414)*
Fasting C-peptide, nmol/l^a^	24	0.58 (0.46–0.70)	19	1.13 (0.98–1.51)*	19	0.63 (0.46–0.70)
C-peptide during OGTT, nmol/l^a^						
Maximum	21	5.89 (4.87–6.79)	17	4.97 (3.44–6.23)	19	3.41 (2.73–3.74)*
Mean	21	2.80 (2.16–3.30)	17	3.38 (2.63–4.08)	19	2.33 (1.79–2.55)*

Data are means ± SD or median (IQR) for pregnant women after RYGB surgery and pregnant control women with non-operated obesity (pre-gestational BMI ≥ 35 kg/m^2^) and normal weight (pre-gestational BMI < 25 kg/m^2^), unless otherwise stated

^a^Rank-based inference was used for comparisons

^b^Early insulin secretion, calculated as AUC_Insulin_/AUC_Glucose 0–60 min_

^c^Late insulin secretion, calculated as AUC_Insulin_/AUC_Glucose 60–180 min_

^d^Total insulin secretion, calculated as AUC_Insulin_/AUC_Glucose 0–180 min_

*p < 0.05 vs RYGB

BMIPG, pre-gestational BMI

### OGTT glucose kinetics during pregnancy

We observed major alterations in glucose kinetics during the OGTT (Fig. 1a–c). There was an early rise in blood glucose in the RYGB group, who reached the highest maximum glucose concentrations, followed by hypoglycaemia (< 2.78 mmol/l) in 19/21 (90%) women with available OGTT data (vs *n* = 0/17 in the obese group and *n* = 1/19 in the normal weight group; *p* < 0.001 for both). In the RYGB group, the mean nadir after oral glucose load reached 2.36 ± 0.66 mmol/l, which was significantly lower than that in the obese group (4.67 ± 0.57 mmol/l; *p* < 0.001) or the normal weight group (3.83 ± 0.67 mmol/l; *p* < 0.001) women. The high glycaemic variability seen in pregnant women after RYGB was also reflected by an increased WHOSH-G (1.30 ± 0.42 for the RYGB group vs 0.78 ± 0.25 for obese women and 0.73 ± 0.32 for normal weight women; *p* < 0.001 for both). Of the participants for which pregnancy OGTT data was available at fasting, 60 and 120 min, seven out of 21 (33%) women who had undergone gastric bypass, seven out of 18 (39%) obese women and none out of 19 normal weight control women met the criteria for GDM diagnosis (fasting glucose ≥ 5.1 mmol/l and/or glucose at 60 min post-load ≥ 10 mmol/l and/or glucose at 120 min post-load ≥ 8.5 mmol/l). All women in the RYGB group had normal fasting glucose and 120 min post-load glucose concentrations < 8.5 mmol/l.

### Insulin secretion and insulin action during pregnancy

**Fig. 2:**
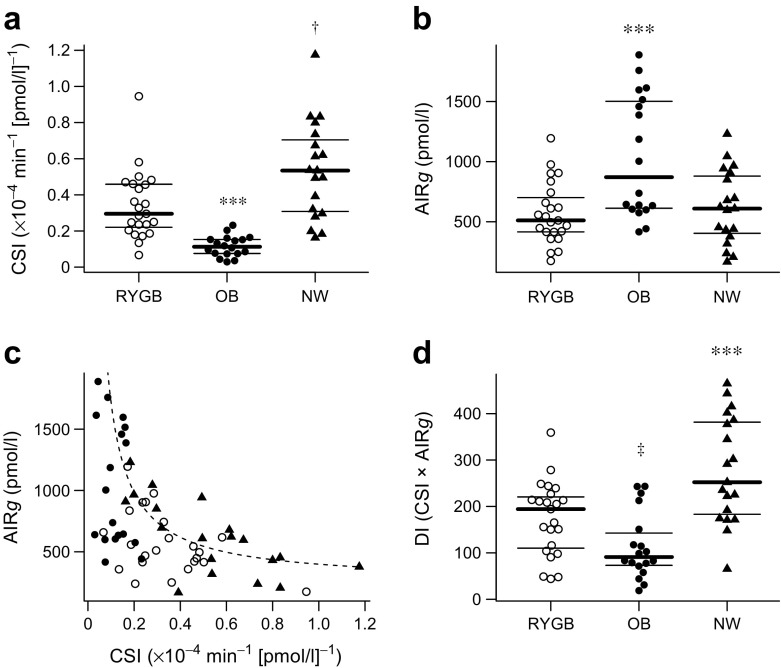
Bee swarm plots, showing data from the IVGTT, representing comparisons of insulin sensitivity (**a**) and insulin secretion (RYGB vs NW, *p* = 0.661) (**b**) and the association between insulin sensitivity and insulin secretion (showing estimated hyperbolic regression line [*y* = 252.3 + *x* ^−1^ × 146.5]) for the control group with normal glucose tolerance (**c**), as well as the DI, representing beta cell function (**d**). RYGB, pregnant women after Roux-en-Y gastric bypass; OB, pregnant women with pre-gestational obesity; NW, pregnant women of normal weight (control group). Bold horizontal line indicates the second (median) quartile, with the lower and upper horizontal lines indicating the first and third quartiles respectively. ***p < 0.001, ^†^ *p* = 0.002, ^‡^ *p* = 0.038 vs RYGB

RYGB mothers showed increased early and total insulin secretion during the OGTT and reached higher maximum concentrations of insulin and C-peptide as compared with the normal weight control group (Table 1), whereas no differences in insulin responses were observed after parenteral glucose administration (mean difference in AIR*g*: −49.5 pmol/l [95% CI −274.5, 175.4]; *p* = 0.661, RYGB vs normal weight). Serum glucagon was notably elevated after oral glucose ingestion in the RYGB group vs obese and normal weight control groups (Fig. 1g–i). Moreover, IVGTT data indicated that the RYGB group had subtle impairment in insulin action (mean difference in CSI: −0.193 × 10^−4^ min^−1^ [pmol/l]^−1^ [95% CI −0.313, −0.073]; *p* = 0.002, RYGB vs normal weight) and beta cell function (mean difference in DI: −103 × 10^−4^ [95% CI −159, −47]; *p* < 0.001, RYGB vs normal weight) (Fig. 2). Of note, when compared with obese mothers, RYGB mothers showed improved insulin sensitivity (mean difference in CSI: 0.226 × 10^−4^ min^−1^ [pmol/l]^−1^ [95% CI 0.104, 0.348]; *p* < 0.001) and a comparable effect was observed for QUICKI and OGIS (Table 1). The differences in IVGTT variables remained unchanged in a sensitivity analysis after excluding women with multiple pregnancies.

### BMI-independent effects on glucose disposal

To further investigate BMI-independent effects on glucose disposal, we matched the RYGB group with a subgroup of the obese and normal-weight women according to BMI at the first visit. A comparison of both groups is provided in Table 2 and revealed that some indices of insulin sensitivity (QUICKI and OGIS) remained significantly improved in the RYGB group, while glucose variability (WHOSH-G) during the OGTT was still increased in those who had previously undergone RYGB.

**Table 2:** Comparison of glucometabolic variables during pregnancy after RYGB and in the BMI-matched control group

Variable	RYGB	BMI-matched control group	*p* value
*n*	14	14	
Age, years	32.9 (28.0–36.5)	33.4 (30.5–35.9)	0.713
BMIV1, kg/m^2^	28.7 (28.0–36.5)	28.8 (27.4–37.7)	0.974
CSI, ×10^−4^ min^−1^ (pmol/l)^−1^	0.404 (0.257–0.480)	0.288 (0.077–0.524)	0.360
AIR*g*, pmol/l	521 (428–712)	773 (627–1151)	0.023
DI (× 10^−4^)	210 (173–243)	229 (91–362)	0.770
QUICKI	0.37 (0.36–0.39)	0.34 (0.32–0.36)	0.021
OGIS, ml min^−1^ m^−2^	520 (474–573)	445 (405–490)	0.005
Insulin secretion during OGTT, pmol/mmol			
Early^a^	106 (79–134)	69 (51–117)	0.125
Late^b^	57 (46–92)	81 (54–165)	0.233
Total^c^	83 (59–110)	79 (52–141)	0.868
Fasting glucose, mmol/l	4.03 (3.95–4.21)	4.42 (4.13–4.66)	0.010
Glucose during OGTT, mmol/l			
Minimum	2.22 (1.96–2.67)	4.34 (3.95–4.66)	< 0.001
Maximum	9.06 (8.58–10.84)	7.87 (6.59–8.80)	0.020
Mean	5.03 (4.69–5.78)	6.48 (5.32–6.85)	0.032
WHOSH-G	1.24 (0.99–1.45)	0.71 (0.48–0.85)	< 0.001

Data are median (IQR) for 14 pregnant women after RYGB and for the 14 pregnant women in the BMI-matched control group (which included *n* = 6 obese and *n* = 8 normal weight women)

^a^Early insulin secretion, calculated as AUC_Insulin_/AUC_Glucose 0–60 min_

^b^Late insulin secretion, calculated as AUC_Insulin_/AUC_Glucose 60–180 min_

^c^Total insulin secretion, calculated as AUC_Insulin_/AUC_Glucose 0–180 min_

All comparisons were based on rank-based inference

BMIV1, BMI at first visit (24–28 weeks of gestation)

### Examination of glucose disposal after delivery

Glucose metabolism was re-examined in a subgroup of women 3–6 months after delivery. Paired comparisons (pregnancy vs postpartum) of women who had undergone RYGB are given in Table 3. Following delivery, the RYGB group showed ameliorated insulin sensitivity and DI (calculated from IVGTT data), as well as increased fasting plasma glucose.

**Table 3:** Glucose metabolism after delivery

Variable	*n*	RYGB V1	RYGB V2	*p* value
CSI, 10^−4^ min^−1^ (pmol/l)^−1^	14	0.345 (0.240–0.460)	0.789 (0.490–0.868)	0.007
AIR*g*, pmol/l	14	435 (373–555)	411 (224–578)	0.280
DI (× 10^−4^)	14	180 (116–213)	243 (197–318)	0.023
QUICKI	19	0.37 (0.36–0.39)	0.39 (0.36–0.44)	0.265
OGIS, ml min^−1^ m^−2^	13	538 (467–578)	546 (500–572)	0.840
Insulin secretion during OGTT, pmol/mmol				
Early^a^	14	106 (83–135)	84 (69–123)	0.244
Late^b^	13	57 (54–97)	40 (25–65)	0.204
Total^c^	13	85 (68–119)	66 (49–115)	0.177
Fasting glucose, mmol/l	19	4.11 (3.95–4.37)	4.45 (4.23–4.67)	< 0.001
Glucose during OGTT, mmol/l				
Minimum	14	2.39 (2.03–2.66)	2.25 (2.06–2.66)	0.941
Maximum	14	8.87 (8.35–9.97)	8.54 (7.77–9.87)	0.312
Mean	14	4.90 (4.69–5.73)	4.74 (4.45–5.13)	0.182
WHOSH-G	14	1.24 (0.94–1.45)	1.18 (0.98–1.52)	0.827

Data are median (IQR) during pregnancy (visit 1 [V1]) and after delivery (visit 2 [V2]) for the RYGB group. The number (*n*) of pairs with available data at both examinations (V1 and V2) is shown

^a^Early insulin secretion, calculated as AUC_Insulin_/AUC_Glucose 0–60min_

^b^Late insulin secretion, calculated as AUC_Insulin_/AUC_Glucose 60–180 min_

^c^Total insulin secretion, calculated as AUC_Insulin_/AUC_Glucose 0–180 min_

All comparisons of metric-scaled variables were based on rank-based inference

Analysis of change scores in women postpartum revealed that CSI showed a greater improvement in the RYGB group when compared with obese women (median [IQR] increase: RYGB, 0.299 × 10^−4^ min^−1^ [pmol/l]^−1^ [0.162–0.561]; obese, 0.068 × 10^−4^ min^−1^ [pmol/l]^−1^ [0.015–0.079]; *p* = 0.002). No significant differences were found between the RYGB group and normal weight women (0.157 × 10^−4^ min ^−1^ [pmol/l] ^−1^, [IQR: 0.030–0.613]; *p* = 0.475). Also, DI was improved, although the improvement did not reach statistical significance, in the RYGB group compared with obese women or normal weight women (median [IQR] increase: RYGB, 92.6 × 10^−4^ [26.0–160.3]; obese, −5.5 [−53–48.2]; normal weight, 9.5 × 10^−4^ [−128.1–127.2]; *p*_overall_ = 0.064).

While fasting plasma glucose increased (pregnancy vs after delivery, Table 3), the postpartum glucose nadir was comparable with that seen in the pregnant state in women who had undergone RYGB, who consequently showed a high risk for hypoglycaemia (< 2.78 mmol/l) after delivery (RYGB *n* = 12/15 [80%] vs obese control women *n* = 0/9 [0%] and vs normal weight *n* = 3/14 [21%]; *p* < 0.001 and *p* = 0.003, respectively). The median (IQR) weight loss after delivery was 10.1 kg (1.5–13.1) in RYGB vs 5 kg (4.0–7.0) (*p* = 0.378) in obese women and 4 kg (1.1–6.3) (*p* = 0.110) in the normal weight control group.

### Neonatal biometry

Neonatal biometry data after delivery was available in 48 women. Offspring born to mothers in the RYGB group had lower mean birthweight percentiles than those born to obese mothers (42.8 ± 29.5 vs 67.1 ± 23.7; *p* = 0.017) and despite mothers in the RYGB group having a higher BMI, no differences were observed between their offspring and those of normal weight mothers (42.0 ± 29.9; *p* = 0.940). For the entire population, birthweight percentiles after delivery were significantly associated with BMI before (ρ = 0.39 [95% CI 0.11, 0.63]; *p* = 0.006) and during pregnancy (ρ = 0.42 [95% CI 0.13, 0.66]; *p* = 0.004), as well as with mean glucose (ρ = 0.36 [95% CI 0.06, 0.60]; *p* = 0.017) and glucose nadir levels during the OGTT (ρ = 0.35 [95% CI 0.03, 0.61]; *p* = 0.019). The relationship between glucose nadir and neonatal growth was stronger in the RYGB group (ρ = 0.89 [95% CI 0.64, 0.97]; *p* < 0.001) and was further underlined by an inverse correlation between neonatal weight and insulin secretion during the OGTT in this group (early secretion, ρ = −0.62 [95% CI −0.88, −0.19]; total secretion, ρ = −0.73 [95% CI −0.94, −0.34]; *p* = 0.017 and *p* = 0.003, respectively). The associations are illustrated in ESM Fig. 2; however, the limited sample size should be considered when interpreting these results.

## Discussion

In this study, we aimed to assess glucometabolic consequences for pregnancy following gastric bypass surgery and observed severely altered glucose regulation, mainly characterised by an early glucose peak and concomitant rise in insulin secretion within the first 60 min following an oral glucose stimulation. Thereby, maximum concentrations of insulin and C-peptide were comparable between the RYGB group and more insulin-resistant women with pre-gestational obesity (obese group), resulting in hypoglycaemia in 90% of mothers who had previously undergone RYGB. Of note, this exaggerated pancreatic insulin response was not observed after parenteral glucose administration (IVGTT-derived insulin response was comparable between the post RYGB and normal weight group) and is therefore likely attributed to the specific anatomical rearrangements after gastric bypass surgery, with possible consequences for pregnancy and pregnancy outcomes.

The disparity between outcomes of OGTT and IVGTT has been observed in previous studies of patients who had previously undergone RYGB and could be explained by the different physiological meanings of these test outcomes [26]; while IVGTTs reflect the sole ‘intrinsic’ effect of plasma glucose on beta cell secretion, OGTTs additionally provide information on potentiating intestinal (‘extrinsic’) mechanisms of insulin release. Indeed, several studies have identified a fundamental role for intestinal incretin peptides, particularly glucagon-like peptide-1 (GLP-1), in modulating pancreatic insulin secretion in non-pregnant patients (men and women) who had undergone RYGB [27–29]. One prominent theory suggests that enhanced GLP-1 release is mainly caused by direct stimulation of the intestinal mucosa following accelerated delivery of undigested nutrients (hindgut hypothesis) [30, 31]. However, the exact mechanisms underlying how the specific gastrointestinal anatomy after RYGB surgery leads to alteration of insulin response are not fully understood; the loss of inhibitory feedback mechanisms owing to bypassing the upper part of the duodenum might be involved (foregut hypothesis) [32–34]. It is not the scope of this report to further investigate this complex interaction between gut and pancreas. Nevertheless, our study design allows a detailed examination of insulin secretion after oral and i.v. glucose load and allows a qualitative interpretation of the postulated ‘extrinsic’ effect on insulin secretion. This effect was increased in the RYGB group during pregnancy (as an exaggerated insulin release was observed only during the OGTT and not during the IVGTT) and likely contributed to the high glycaemic variability seen after oral glucose administration. In line with our results, a recent study that used continuous subcutaneous glucose monitoring to evaluate glucose profiles in 35 bariatric pregnancies, suggested that abnormal glucose variability occurred in real-life conditions [35]. It is also worth noting that we found an exaggerated glucagon release immediately after glucose ingestion in the RYGB group. Although supported by other studies in the literature (e.g. [36]), this is somewhat paradoxical when considering the concomitant rise in plasma glucose and its inhibitory effect on alpha cell function [37]. Whether this observation represents a physiological adaptation for maintaining euglycaemia [38], or rather reflects an increased release of inactive proglucagon because of stimulation of intestinal L cells interfering with glucagon assays [26], is debatable.

Pancreatic insulin secretion is strongly related to the extent of insulin action in different target tissues (i.e. mainly liver and skeletal muscle). Consequently, altered insulin secretion and its possible impact on glucose kinetics following RYGB surgery need to be interpreted in relation to the specific degree of insulin resistance. In this context, we observed an improvement in fasting and dynamic indices of insulin action at 24–28 weeks of gestation in women post RYGB vs obese pregnant women. In addition, analysis of IVGTT data revealed subtle alterations in insulin sensitivity and beta cell function during pregnancy in the RYGB group vs the normal weight control group, which could be explained by their higher degree of pre-gestational overweight and obesity. However, it may be important that fasting (hepatic) and dynamic insulin sensitivity during the OGTT (representing whole-body insulin action) remained significantly improved in the RYGB group vs the BMI-matched control group, indicating that bariatric surgery also has body-weight-independent effects on glucose disposal during gestation. This notion is also supported by studies in non-pregnant women post RYGB (for detailed reviews, see [26, 30]), mainly suggesting that improved fasting insulin sensitivity, which is already evident in the first week after gastric bypass, is related to energy restriction and reduced liver fat content rather than to changes in body weight. Such an early improvement in insulin sensitivity is not observed during the hyperinsulinaemic–euglycaemic clamp (characterising insulin action in the skeletal muscle), where improvement occurs months after surgery, together with ongoing weight loss [36]. Hence, we conclude that the degree of insulin sensitivity in pregnant women after RYGB seems to be a consequence of at least two mechanisms: energy restriction and the amount of weight lost after surgery. However, it must be mentioned that the action of insulin changes during gestation [39]; in normal pregnancy insulin sensitivity is unchanged or improved in early gestation, followed by a constant decrease (up to 60%) until the third trimester. Hence, it might be a limitation of our study that women were examined only once during gestation. Nevertheless, owing to a lack of information on this topic our study represents the first attempt to assess the underlying physiological mechanisms associated with bariatric surgery during pregnancy. We decided to perform our examinations in accordance with the timing of standard GDM screening, although it is actually unclear how the physiological adaptation of glucose homeostasis during pregnancy interacts with disturbed glucose disposal after gastric bypass and this needs to be addressed in future investigations.

We observed that insulin sensitivity and DI (using IVGTT data) was improved in the RYGB group after delivery. Of note, the improvement in IVGTT-derived insulin sensitivity was greater in the RYGB group than in obese women. This was somewhat unexpected but might be related to the metabolic effects of gastric bypass surgery (as discussed above) or to several changes that interact with insulin sensitivity in the postpartum period (such as weight loss, dietary patterns and breast feeding). The weight change did not differ significantly between the groups in our study. However, it has to be mentioned as a limitation that several participants dropped out of the study at the postpartum visit and so this issue needs further investigation. The postpartum glucose nadir during the OGTT and risk for hypoglycaemia in the RYGB group were comparable with our findings at 24–28 weeks of gestation.

Further consequences for pregnancy and pregnancy outcomes after RYGB surgery need to be addressed. First, one must question the reliability of recommended diagnosis criteria (e.g. [40]) for identifying or ruling out hyperglycaemia in pregnancies following gastric bypass. In our study, the RYGB group had improved fasting glucose despite higher pre-gestational body weight; however, they showed increased peak glucose concentrations after oral ingestion, mostly at a median time of 30 min. It is not clear whether the standard OGTT, which includes glucose measurements at fasting and at 60 and 120 min after ingestion of glucose, can adequately reflect a rise in plasma glucose before 60 min. Second, the increased risk for hypoglycaemia during later periods of the OGTT is another important limitation of this test. We recently examined glucose profiles during a 2 h OGTT screening for GDM in a larger but retrospective sample of women after gastric bypass and observed that hypoglycaemia (< 3.34 mmol) occurred in more than 50% of individuals [41]. This is in accordance with our current study, providing further evidence to support the consideration of postprandial hypoglycaemia as a frequent and potentially harmful side-effect of OGTT screening in pregnancy after RYGB. Third, some studies have reported an increased incidence of SGA offspring after gastric bypass surgery as well as a trend towards higher perinatal morbidity [13, 14, 41]. Therefore, our finding that fetal growth of RYGB offspring was associated with the glucose nadir during the OGTT might be of importance, possibly suggesting that the higher level of glycaemic variability and incidence of postprandial hypoglycaemia after gastric bypass has an impact on fetal development. While the restricted sample size of our study must be noted, this association indicates the need for further examinations containing continuous longitudinal glucose profiles during pregnancy, providing detailed information on glucose variability, duration of hyper- and hypoglycaemic episodes, and variables of glucose disposal and their relation to fetal growth.

In summary, we observed that glucose metabolism is notably altered in pregnancies after gastric bypass. This is due to changes in insulin action and exaggerated insulin release early after ingestion of glucose (mainly a consequence of a strong interaction between the gut and pancreas), potentially affecting glucose kinetics during the OGTT or in real-life conditions. These physiological changes could have potential implications for pregnancy, indicating the need for further longitudinal investigations.

## Electronic supplementary material

ESM (PDF 252 kb)

## Abbreviations

AIR*g*: Acute insulin response to glucose
CSI: Calculated sensitivity index
DI: Disposition index
GDM: Gestational diabetes mellitus
GLP-1: Glucagon-like peptide-1
IQR: Interquartile range
LGA: Large for gestational age
OGIS: Oral glucose insulin sensitivity index
QUICKI: Quantitative insulin sensitivity check index
RYGB: Roux-en-Y gastric bypass
SGA: Small for gestational age
WHOSH-G: Shape index of glucose

## References

[1] Jia H, Lubetkin EI (2005). The impact of obesity on health-related quality-of-life in the general adult US population. J Public Health.

[2] Bray GA (2004). Medical consequences of obesity. J Clin Endocrinol Metab.

[3] Buchwald H, Consensus Conference Panel (2005). Consensus conference statement bariatric surgery for morbid obesity: health implications for patients, health professionals, and third-party payers. Surg Obes Relat Dis.

[4] Colquitt JL, Pickett K, Loveman E, Frampton GK (2014) Surgery for weight loss in adults. Cochrane Database Syst Rev (8):CD003641 10.1002/14651858.CD003641.pub4 PMC9028049 25105982

[5] Carlsson LM, Peltonen M, Ahlin S (2012). Bariatric surgery and prevention of type 2 diabetes in Swedish obese subjects. N Engl J Med.

[6] Mingrone G, Panunzi S, De Gaetano A (2012). Bariatric surgery versus conventional medical therapy for type 2 diabetes. N Engl J Med.

[7] Poston L, Caleyachetty R, Cnattingius S (2016). Preconceptional and maternal obesity: epidemiology and health consequences. Lancet Diabetes Endocrinol.

[8] Weintraub AY, Levy A, Levi I, Mazor M, Wiznitzer A, Sheiner E (2008). Effect of bariatric surgery on pregnancy outcome. Int J Gynaecol Obstet.

[9] Bennett WL, Gilson MM, Jamshidi R (2010). Impact of bariatric surgery on hypertensive disorders in pregnancy: retrospective analysis of insurance claims data. BMJ.

[10] Roos N, Neovius M, Cnattingius S (2013). Perinatal outcomes after bariatric surgery: nationwide population based matched cohort study. BMJ.

[11] Kjær MM, Lauenborg J, Breum BM, Nilas L (2013). The risk of adverse pregnancy outcome after bariatric surgery: a nationwide register-based matched cohort study. Am J Obstet Gynecol.

[12] Adams TD, Hammoud AO, Davidson LE (2015). Maternal and neonatal outcomes for pregnancies before and after gastric bypass surgery. Int J Obes.

[13] Lesko J, Peaceman A (2012). Pregnancy outcomes in women after bariatric surgery compared with obese and morbidly obese controls. Obstet Gynecol.

[14] Johansson K, Cnattingius S, Näslund I (2015). Outcomes of pregnancy after bariatric surgery. N Engl J Med.

[15] Goldfine AB, Mun EC, Devine E (2007). Patients with neuroglycopenia after gastric bypass surgery have exagerated incretin and insulin secretory responses to a mixed meal. J Clin Endocrinol Metab.

[16] Ho DE, Imai K, King G, Stuart EA (2011). MatchIt: nonparametric preprocessing for parametric causal inference. J Stat Softw.

[17] Tura A, Morbiducci U, Sbrignadello S, Winhofer Y, Pacini G, Kautzky-Willer A (2011). Shape of glucose, insulin, C-peptide curves during a 3-h oral glucose tolerance test: any relationship with the degree of glucose tolerance?. Am J Phys Regul Integr Comp Phys.

[18] Katz A, Nambi SS, Mather K (2000). Quantitative insulin sensitivity check index: a simple, accurate method for assessing insulin sensitivity in humans. J Clin Endocrinol Metab.

[19] Tura A, Sbrignadello S, Succurro E, Groop L, Sesti G, Pacini G (2010). An empirical index of insulin sensitivity from short IVGTT: validation against the minimal model and glucose clamp indices in patients with different clinical characteristics. Diabetologia.

[20] Kahn SE, Prigeon RL, McCulloch DSK (2002). Quantification of the relationship between insulin sensitivity and β-cell function in human subjects: evidence for a hyperbolic function. Diabetes.

[21] Mari A, Pacini G, Murphy E, Ludvik B, Nolan JJ (2001). A model based method for assessing insulin sensitivity from the oral glucose tolerance test. Diabetes Care.

[22] Tura A, Kautzky-Willer A, Pacini G (2006). Insulinogenic indices from insulin and C-peptide: comparison of beta-cell function from OGTT and IVGTT. Diabetes Res Clin Pract.

[23] Brunner E, Munzel U (2000). The nonparametric Behrens-Fisher Problem: asymptotic theory and a small-sample approximation. Biom J.

[24] Neubert K, Brunner E (2007). A studentized permutation test for the nonparametric Behrens-Fisher Problem. Comput Stat Data Anal.

[25] R Core Team. R. A language and environment for statistical computing. R Foundation for Statistical Computing 2016, Vienna, Austria. Available from http://www.R-project.org/. Accessed 19 February 2017

[26] Dirksen C, Jørgensen NB, Bojsen-Møller KN (2012). Mechanisms of improved glycaemic control after Roux-en-Y gastric bypass. Diabetologia.

[27] Laferrère B, Heshka S, Wang K (2007). Incretin levels and effect are markedly enhanced 1 month after Roux-en-Y gastric bypass surgery in obese patients with type 2 diabetes. Diabetes Care.

[28] Anderwald CH, Tura A, Promintzer-Schifferl M (2012). Alterations in gastrointestinal, endocrine, and metabolic processes after bariatric Roux-en-Y gastric bypass surgery. Diabetes Care.

[29] Salehi M, Prigeon RL, D'Alessio DA (2011). Gastric bypass surgery enhances glucagon-like peptide 1-stimulated postprandial insulin secretion in humans. Diabetes.

[30] Mosinski JD, Kirwan JP (2016). Longer-term physiological and metabolic effects of gastric bypass surgery. Curr Diab Rep.

[31] Holst JJ, Madsbad S (2016). Mechanisms of surgical control of type 2 diabetes: GLP-1 is key factor. Surg Obes Relat Dis.

[32] Rubino F, Gagner M (2002). Potential of surgery for curing type 2 diabetes mellitus. Ann Surg.

[33] Kamvissi V, Salerno A, Bornstein SR, Mingrone G, Rubino F (2015). Incretins or anti-incretins? A new model for the “entero-pancreatic axis”. Horm Metab Res.

[34] Vidal J, de Hollanda A, Jiménez A (2016). GLP-1 is not the key mediator of the health benefits of metabolic surgery. Surg Obes Relat Dis.

[35] Bonis C, Lorenzini F, Bertrand M (2016). Glucose profiles in pregnant women after a gastric bypass: findings from continuous glucose monitoring. Obes Surg.

[36] Bojsen-Møller KN, Dirksen C, Jørgensen NB (2014). Early enhancements of hepatic and later of peripheral insulin sensitivity combined with increased postprandial insulin secretion contribute to improved glycemic control after Roux-en-Y gastric bypass. Diabetes.

[37] Holst JJ (2011). Postprandial insulin secretion after gastric bypass surgery: the role of glucagon-like peptide 1. Diabetes.

[38] Camastra S, Muscelli E, Gastaldelli A (2013). Long-term effects of bariatric surgery on meal disposal and β-cell function in diabetic and nondiabetic patients. Diabetes.

[39] Barbour LA, McCurdy CE, Hernandez TL, Kirwan JP, Catalano PM, Friedman JE (2007). Cellular mechanisms for insulin resistance in normal pregnancy and gestational diabetes. Diabetes Care.

[40] International Association of Diabetes and Pregnancy Study Groups Consensus Panel (2010). International association of diabetes and pregnancy study groups recommendations on the diagnosis and classification of hyperglycemia in pregnancy. Diabetes Care.

[41] Feichtinger M, Stopp T, Hofmann S (2017). Altered glucose profiles and risk for hypoglycaemia during oral glucose tolerance testing in pregnancies after gastric bypass surgery. Diabetologia.

