# Supplementary material for: Assessment of glucose regulation in pregnancy after gastric bypass surgery
Source: Diabetologia. 2017 Sep 16;60(12):2504–13. doi: 10.1007/s00125-017-4437-6 (PMC6448941; doi:10.1007/s00125-017-4437-6)

ESM Methods: Data imputations. Multivariate imputations were performed for missing OGTT and IVGTT data if at least 50% of the measurements of the respective experiment was available.

	**Glucose OGTT**	**Insulin OGTT**	**C-Peptide OGTT**	**Glucagon OGTT**
Time of OGTT sampling	*Number of Imputations*			
**0'**	0	0	0	1
**30'**	0	0	0	1
**60'**	0	0	0	0
**90'**	0	0	0	2
**120'**	0	0	0	0
**150'**	6	5	5	5
**180'**	6	6	6	6
**Total number of imputations**	12	11	11	15

	**Glucose IVGTT**	**Insulin IVGTT**	**C-Peptide IVGTT**	**- -**
Time of OGTT sampling	*Number of Imputations*			
**0'**	0	1	1	
**3'**	2	2	2	
**4'**	0	0	0	
**5'**	0	0	0	
**6'**	0	0	0	
**8'**	0	0	0	
**10'**	0	0	0	
**15'**	0	0	0	
**20'**	1	0	0	
**30'**	1	2	2	
**40'**	0	1	0	
**50'**	0	0	0	
**60'**	1	0	1	
**Total number of imputations**	5	6	6	

ESM Fig1: Available and missing OGTT and IVGTT examinations.

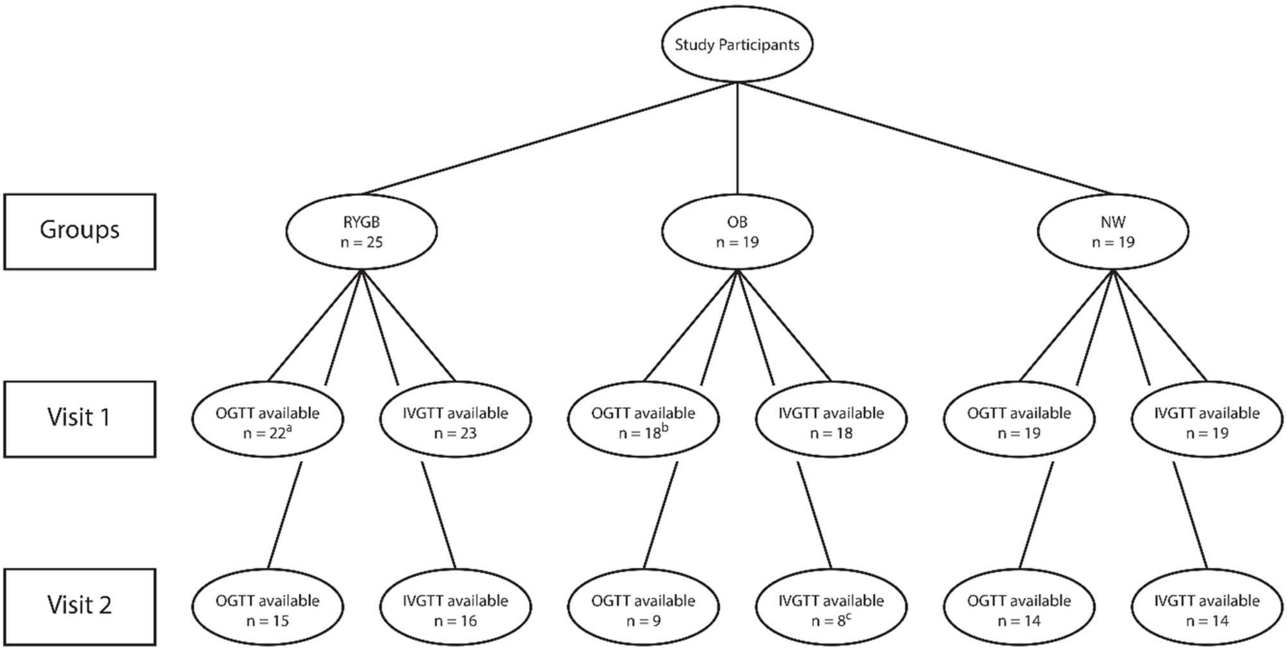

[a]In one women the OGTT was discontinued at 60 min. Imputations were not used as >50% of the data were missing. Data of this test were included in summary statistics of fasting values (including QUICKI) and early insulin secretion but not included in summary statistics of other dynamic OGTT indices, status of GDM or hypoglycemia.

[b]In one women only measurements at fasting, 60 and 120 min were available. Imputations were not used as >50% of the data were missing. Data of this test were included in summary statistics of fasting values (including QUICKI) and GDM status, but not included in summary statistics of other dynamic OGTT indices or hypoglycemia.

[c]In one women only glucose values of this test were available (values of insulin and C-peptide were missing) and calculation of CSI and AIRg was not possible.

Additional comments: In one RYGB women OGTT and IVGTT was missing at visit 1 and only postpartum IVGTT data and data on fetal biometry was available. The OGTT was discontinued in six RYGB women (1×Visit 1; 5×Visit 2) due to side-effects like hypoglycemia or vomiting. 18 women (RYGB: 5; OB: 9; NW: 4) did not agree to participate at the postpartum visit.

ESM Fig 2: Association of birth weight percentiles with OGTT early insulin secretion (a), total insulin secretion (b) and glucose nadir (c) after rank transformation in women after gastric bypass surgery.

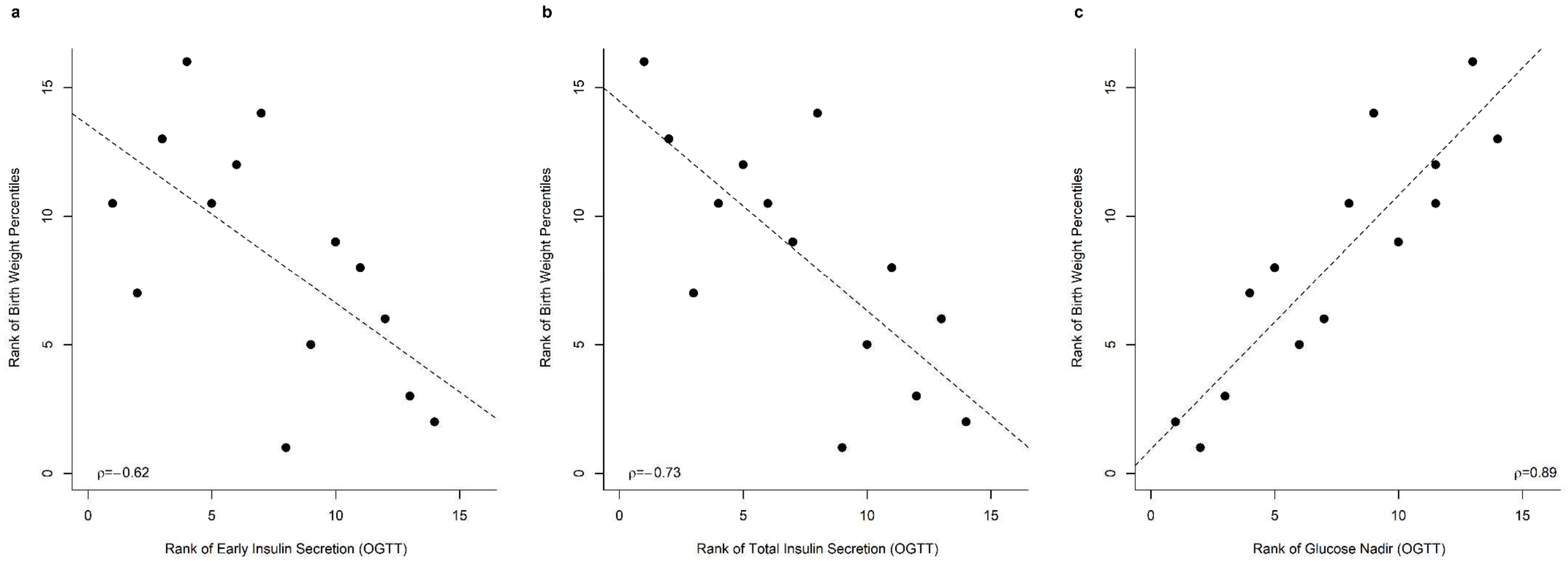

Supplement: Supplementary file 1 — (PDF 252 kb) [file 125_2017_4437_MOESM1_ESM.pdf]
